# 4412 Unraveling the role of the interaction between enteric virus and commensal bacteria in a physiological relevant model of human intestinal epithelium

**DOI:** 10.1017/cts.2020.103

**Published:** 2020-07-29

**Authors:** Carmen Mirabelli

**Affiliations:** 1 University of Michigan School of Medicine

## Abstract

OBJECTIVES/GOALS: In the crowded environment of the intestine, selected commensal bacteria and enteric viruses interact. The biological significance of this interaction, in either normal or pathological condition is not known. To study this interaction, we are developing a physiologically relevant model of an human intestinal epithelium. METHODS/STUDY POPULATION: Intestinal biopsies (ileum region) and fecal samples of 6 healthy and 6 active Crohn’s patients are being collected to derive human intestinal enteroid (HIE) lines. 2D-polarized HIE will be first characterized with studies of epithelial permeability, tight junctions and cell type composition, and co-cultured with matching fecal samples. The (co-)cultures will be then infected with human norovirus (HNoV), our model enteric virus, and infection will be quantified by RT-qPCR. In addition, the interaction of HNoV with bacteria derived from healthy or Crohn’s will be determined quantitatively by flow cytometry (viral tagging) and qualitatively by 16S sequencing of the total *versus* HNoV-bound bacterial species. RESULTS/ANTICIPATED RESULTS: Crohn’s patients are characterized by a microbiome dysbiosis and, in particular, by a high abundance of *Enterobacteriacae*. HNoV interacts with *Enterobacter cloacae*, and interestingly, HNoV infection is associated with exacerbation and reactivation of Crohn’s disease. By re-creating the intestinal milieu of healthy and Crohn’s patients, we expect that the kinetics of infection by HNoV will be higher in Crohn’s as compared to healthy volunteers. In addition, by studying the composition of the HNoV-bound bacterial component of Crohn’s versus heathy volunteers, we will be able to identify the contribution of selected bacteria to the expected increase of infection. DISCUSSION/SIGNIFICANCE OF IMPACT: With this study, we will fill the gap of knowledge on the importance of commensal bacteria and enteric virus interactions in healthy and diseased condition. This new knowledge will be paramount for the identification of novel strategies to combat highly prevalent virus infections.

